# Low-dose versus standard-dose computed tomography-guided biopsy for pulmonary nodules: a randomized controlled trial

**DOI:** 10.1186/s13019-023-02183-8

**Published:** 2023-03-16

**Authors:** Er-Liang Li, Ai-Li Ma, Tao Wang, Yu-Fei Fu, Han-Yang Liu, Guang-Chao Li

**Affiliations:** 1 Department of Radiology, Xuzhou Central Hospital, Xuzhou, China; 2 Department of Interventional Radiology, Xuzhou Central Hospital, Xuzhou, China; 3 Department of Radiology, Shanghai Sixth People’s Hospital, Shanghai, China

**Keywords:** Low-dose, Computed tomography, Biopsy, Lung nodule

## Abstract

**Background:**

To assess relative safety and diagnostic performance of low- and standard-dose computed tomography (CT)-guided biopsy for pulmonary nodules (PNs).

**Materials and methods:**

This was a single-center prospective randomized controlled trial (RCT). From June 2020 to December 2020, consecutive patients with PNs were randomly assigned into low- or standard-dose groups. The primary outcome was diagnosis accuracy. The secondary outcomes included technical success, diagnostic yield, operation time, radiation dose, and biopsy-related complications. This RCT was registered on 3 January 2020 and listed within ClinicalTrials.gov (NCT04217655).

**Results:**

Two hundred patients were randomly assigned to low-dose (n = 100) and standard-dose (n = 100) groups. All patients achieved the technical success of CT-guided biopsy and definite final diagnoses. No significant difference was found in operation time (n = 0.231) between the two groups. The mean dose-length product was markedly reduced within the low-dose group compared to the standard-dose group (31.5 vs. 333.5 mGy-cm, P < 0.001). The diagnostic yield, sensitivity, specificity, and accuracy of the low-dose group were 68%, 91.5%, 100%, and 94%, respectively. The diagnostic yield, sensitivity, specificity, and accuracy were 65%, 88.6%, 100%, and 92% in the standard-dose group. There was no significant difference observed in diagnostic yield (P = 0.653), diagnostic accuracy (P = 0.579), rates of pneumothorax (P = 0.836), and lung hemorrhage (P = 0.744) between the two groups.

**Conclusions:**

Compared with standard-dose CT-guided biopsy for PNs, low-dose CT can significantly reduce the radiation dose, while yielding comparable safety and diagnostic accuracy.

## Background

In recent years, computed tomography (CT) screening for lung cancer has become a routine examination [1–3]. Therefore, the detection rate of pulmonary nodules (PNs) has also increased. However, according to the guidelines for the management of incidental PNs on CT, no routine follow-up or optional CT at 12 months is recommended for PNs ≤ 5 mm. This is because the chances of malignancy are very low in nodules ≤ 5 mm in size [4]. However, the malignancy rate ranges from 43 to 63% when the PNs > 5 mm [5, 6]. CT-guided biopsy is commonly employed for differential diagnosis in PNs due to its advantages, such as simplicity and minimal invasiveness, with the diagnostic accuracy of 92.7–97.0% [6–8].

Compared to CT-guided biopsy for lung masses, biopsy for PNs is a more difficult technique due to the smaller lesion size. Therefore, when performing CT-guided biopsy for PNs, prolonged CT scans are usually required to adjust the needle position and angle [9–11]. Consequently, exposing individual patients to additional radiations. Therefore, low-dose CT procedures were employed to decrease unnecessary dosing during CT-guided interventions [11]. Previous investigations have also described low-dose CT-guided biopsy for diagnosing PNs [9, 10]. However, these studies were retrospective with a high risk of selection, comparability, and outcome bias. A previous randomized controlled trial (RCT) has been conducted to assess the relative safety and diagnostic accuracy between low-dose and standard-dose CT-guided lung biopsy [12]. However, that RCT contained both lung masses and LNs [12], and thus, the outcomes of low-dose CT-guided biopsy for PNs are still unclear. Therefore, a well-designed RCT which only focuses on low-dose CT-guided biopsy for PNs should be conducted.

In this study, we conducted an RCT to evaluate the relative safety and diagnostic performance of low- and standard-dose CT-guided biopsy for PNs.

## Methods

### Study design

The study protocol of this single-center RCT was approved by our center’s Institutional Review Board. All participants gave written, informed-consent. In addition, this RCT was listed within ClinicalTrials.gov (NCT04217655).

From June 2020 to December 2020, consecutive eligible patients with PNs were randomly assigned into low-dose and standard-dose groups. Table 1 showed the scanning parameters of the low-dose and standard-dose CT.

**Table 1:** Scanning parameters between 2 groups

	Low-dose group	Standard-dose group
Tube voltage	120 kV	120 kV
Tube current	15 mA	150 mA
Thickness	2 mm	2 mm
Collimation	16 × 0.75 mm	16 × 0.75 mm
Pitch	1.063	1.063
Rotation time	0.5 s	0.5 s
Field of view	350 mm	350 mm

Inclusion criteria: (a) clinical cases with PNs, detected on CT; (b) solid PNs; (c) PNs > 8 mm; (d) PNs having intermediate-high risk for lung cancer, depending upon clinical/radiology-based characteristics [4].

Exclusion criteria: (a) patients who underwent CT-guided biopsy previously; (b) PNs which were stable in size for at least 1 year; (c) PNs that decreased in size during follow-up; (d) clinical cases having a history of intense cardiac, pulmonary, renal or coagulation dysfunctional conditions; and (e) patients who refused to join this RCT.

The primary endpoint of this RCT was diagnostic accuracy. The secondary endpoints included technical success, diagnostic yield, operation time, radiation dose, and biopsy-related complications.

### Randomization and blinding

The eligible patients were randomly assigned into 1:1 low-dose and standard-dose groups through the block-randomization technique (block size: 8). The randomized computer-generated numbers were placed within sequentially-numbered, opaque, sealed envelopes. Before the biopsy, envelopes were opened by a member of the Science and Education department without a defined role in the trial. This RCT was single-blinded for the patients.

### CT-guided biopsy protocols

All procedures were performed under the guidance of a 16-row CT (Philips™, Cleveland, OH, USA) operated through a CT-guided interventional radiology expert (10+ years). Only the spiral CT was used for guiding the biopsy procedures, while the CT fluoroscopy was not used.

The patient’s position was decided according to the sites of PNs. The needle-paths were chosen depending upon preoperative CT outcomes. The co-axial technique was employed during the procedure. First, a 17G outer needle (DuoSmart™, Modena, Italy) was used to pierce the lung-parenchyma, followed by a second CT scan to establish a needle-tip to displace it accordingly. Once the outer needle-tip touched the PN, an 18G inner semi-automatic core-needle (Wego™, Weihai, China) was inserted via the outer needle to obtain the samples from the PNs. A total of 3–4 samples were obtained from each PN and consequently submerged into 10% formaldehyde until pathology assessment was done.

After biopsy, another CT intervention was conducted to assess the procedure-associated complications.

### Image reconstruction

CT raw data were reconstructed by a third-generation image reconstruction technique (iDose, Philips, Hybrid Model Based Iterative Reconstruction) with strength level of 5, slice thickness of 2 mm, and an increment of 1 mm. Reconstructions using a sharp reconstruction filter (Y-sharp) for lung structures and a standard reconstruction filter (B) for soft tissue structures.

### Imaging subjectivity

Two radiologists (T.W. and E-L.L.) evaluated imaging standards independently. One radiologist (T.W.) had 15 years of experience in CT-guided intervention and the other (E-L.L.) had 8 years of experience in CT-guided intervention. Imaging-quality was evaluated across four categories according to the previous study for low-dose CT-guided lung biopsy [12]: category A: needle/PN were distinctly observable; category B: needle/PN were adequately observable; category C: needle/PN were only somewhat observable; and category D: needle/PN could not be seen. Therefore, only category A and B could be used for CT-guided biopsy procedures. In the case of category C or D images, tube voltage and/or current were adjusted to obtain higher quality images. However, the procedures should be considered a technical failure.

### Evaluation of radiation dose

The radiation dose was assessed by the dose-length product (DLP) value. DLP was measured in mGy*cm and it was a measure of CT tube radiation output/exposure. DLP accounts for the length of the radiation output along the z-axis.

### Definitions and diagnoses

PN was defined as a spherical/oval image of non-transparent lesions ≤ 3 cm in diameter with neighboring pulmonary parenchyma/non-linked to atelectasis, mediastinal lymphadenopathy, or pleural effusion [4]. Technical success for CT-guided biopsy was confirmed once pathologists finalised their diagnosis from extracted specimens [12, 13]. Biopsy-based diagnoses could be classified into four categories: (a) malignancy; (b) suspected malignancy; (c) specific benignity; and (d) non-specific benignity. Suspected malignancy was defined as atypical cells suspected of indicated malignancy [14]. Specific benignity was defined as dataset outcomes suggested defined benign-diagnosis, including hamartomas and tuberculosis [14]. Finally, non-specific benignity was defined as benign pathology characteristics that existed through and did not suffice for a formal diagnosis [14].

Resection was used to make the final diagnoses for both malignant and benign PNs. biopsy-based malignancy and specific benignity could be accepted as the final diagnosis [8–13]. Biopsy-based suspected malignancy and non-specific benignity could not be accepted as the final diagnoses, if they were not confirmed by resection, the CT medical observation would be useful for attaining a finalized diagnostic outcome. For an PN with ≥ 20% size-reduction (without anticancer treatments), or maintained dimensions (no change or decreased < 20%) for a 12 month-minimum period (with no anti-cancer treatments), the final benign diagnosis could be accepted [6, 14].

True-positive was postulated when biopsy-based malignancy/suspicious was confirmed as malignant at finalized-diagnosis. The true-negative was postulated when biopsy-based benignities confirmed benignities at finalized-diagnosis.

Diagnosis yield = (biopsy-based malignancy + biopsy-based specific benignity)/all cases. Diagnostic accuracy = (true positive + true negative)/all cases with the final diagnosis. Pneumothorax and lung hemorrhage were assessed by chest CT. Lung hemorrhage was considered as a novel consolidating/ground-glass opacity around the needle tract [15]. High-grade hemorrhage was defined as the width of needle tract hemorrhage > 2 cm [15].

### Statistical analyses

The sample size was calculated based on diagnostic accuracy with the non-inferiority analysis. Based upon past investigations linked to CT-guided biopsy for PNs, we estimated that the diagnostic accuracy was 94% [6, 8, 12]. Based on the − 10% of non-inferiority margin with the one-sided significance category of 0.025, we estimated that 200 patients (100 patients per group) were needed after considering the 10% dropout rate.

Intention-to-treat (ITT) evaluations were performed depending on the total patient group quantity enrolled in this study. In contrast, per-protocol (PP) evaluations were performed depending on the total number of patients who achieved technical success and definite final-diagnoses.

The continuous data were compared through the independent sample t test when the distribution was normal, while Mann–Whitney U test was used if the distribution was not normal. Categorical data were compared through Pearson χ^2^/Fisher exact test. Univariate and multivariate logistic regression tests were used for predictive indicators of diagnosis accuracy and complications. Kappa analysis was conducted to assess inter-observer agreement regarding imaging-quality. All statistical analyses were conducted through SPSS® v.16.0 (SPSS Inc™, Chicago, Illinois, USA). The significance level was P < 0.05.

## Results

### Patients

**Fig. 1:**
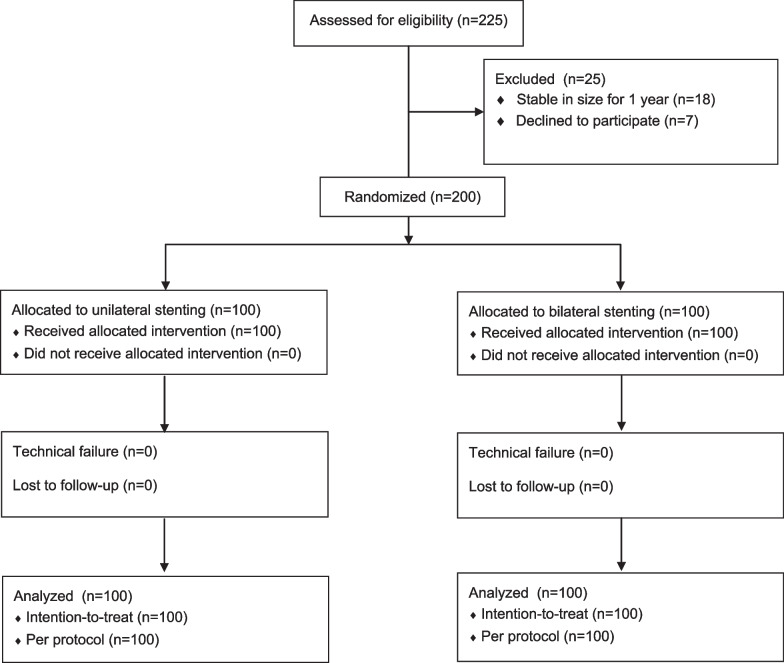
The flowchart of this study

A total of 200 patients who fulfilled the inclusion criteria were randomly assigned to low-dose (n = 100) and standard-dose (n = 100) groups (Fig. 1). All patients achieved the technical success of CT-guided biopsy and the definite final diagnoses. Therefore, ITT and PP analyses were conducted based on the same population (Table 2).

**Table 2:** Baseline data and procedure details between 2 groups

	Low-dose group (n = 100)	Standard-dose group (n = 100)	P value
*Normal data*			
Age (y)	63.7 ± 10.5	61.1 ± 13.0	0.144
Gender (male/female)	68/32	62/38	0.374
Smoking history	49	40	0.200
Tumor history	7	10	0.447
BMI (kg/m^2^)	23.1 ± 3.6	22.8 ± 3.2	0.590
*Imaging findings*			
Emphysema	28	33	0.229
Lesion size (mm)	24.8 ± 4.2	23.5 ± 5.0	0.045
Lung (left/right)	42/58	45/55	0.669
Lobe (upper/non-upper)	45/55	42/58	0.669
*Biopsy procedure*			
Lesion-pleura distance (< / ≥ 30 mm)	85/15	75/25	0.077
Needle-pleura angle (< / ≥ 50 degrees)	13/87	11/89	0.663
Prone/Supine/Decubitus	67/30/3	54/44/2	0.120
Number of needle pathways	1.3 ± 0.7	1.3 ± 0.7	0.694
Number of samples	3.3 ± 0.5	3.3 ± 0.5	0.880
Duration of procedure (min)	10.8 ± 4.1	11.6 ± 5.3	0.231
*Complications*			
Pneumothorax (chest tube insertion)	14 (4)	13 (3)	0.836
Lung hemorrhage (high-grade hemorrhage)	24 (14)	26 (14)	0.744
*Radiation dose*			
DLP (mGy-cm)	31.5 (Q1: 27.3; Q3: 38.2)	333.5 (Q1: 273.6; Q3: 407.9)	< 0.001*

*BMI* body mass index, *DLP* dose-length product

*Mann–Whitney U test results

### Procedure details

Table 2 shows the procedure details of low-/standard-dose CT-guided biopsy. There were no significant differences in the number of needle pathways (n = 0.694), number of samples (P = 0.880), and duration of procedures (n = 0.231) between the two groups. The median DLP was significantly reduced within the low-dose group in comparison to the standard-dose group (P < 0.001).

### Imaging-quality

Within the low-dose group, 72 images (72%) were considered as category A and 28 images (28%) were considered as category B. Within the standard-dose group, all imaging scans were considered as category A. The inter-observer agreements were very good across both groups (kappa value = 0.926 and 1.000, respectively). The category A images occurred more frequently within the standard-dose group (P < 0.001).

### Diagnosis

**Fig. 2:**
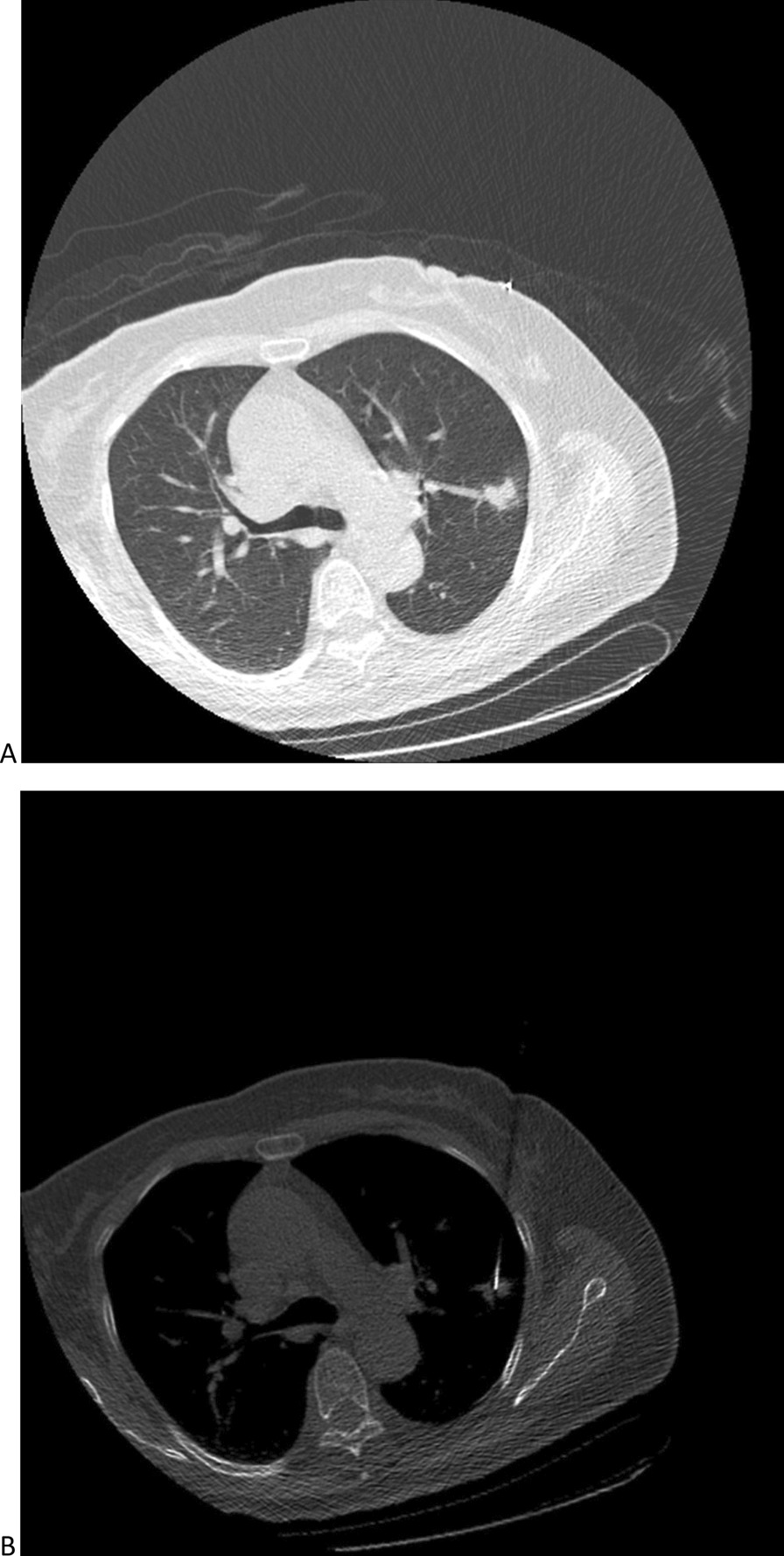
The images for low-dose CT-guided biopsy for PN. **a** Preoperative CT for the PN; **b** the procedure of low-dose CT-guided biopsy

In low-dose group (Fig. 2), biopsy-based diagnoses included malignancy (n = 64), suspicious malignancy (n = 1), specific benignity (n = 4), and non-specific benignity (n = 31). Among them, malignancy/specific benignity results could be approved as finalized-diagnoses. Suspicious malignancy was further validated as lung adenocarcinoma through surgical resection. Twenty-five non-specific benignities were confirmed as true benignities by CT medical observation (n = 23) or surgical resection (n = 2). In contrast, 6 non-specific benignities were confirmed as false benignities by surgical resection. Therefore, the diagnostic yield, sensitivity, specificity, and diagnostic accuracy were 68%, 91.5%, 100%, and 94%, respectively.

**Fig. 3:**
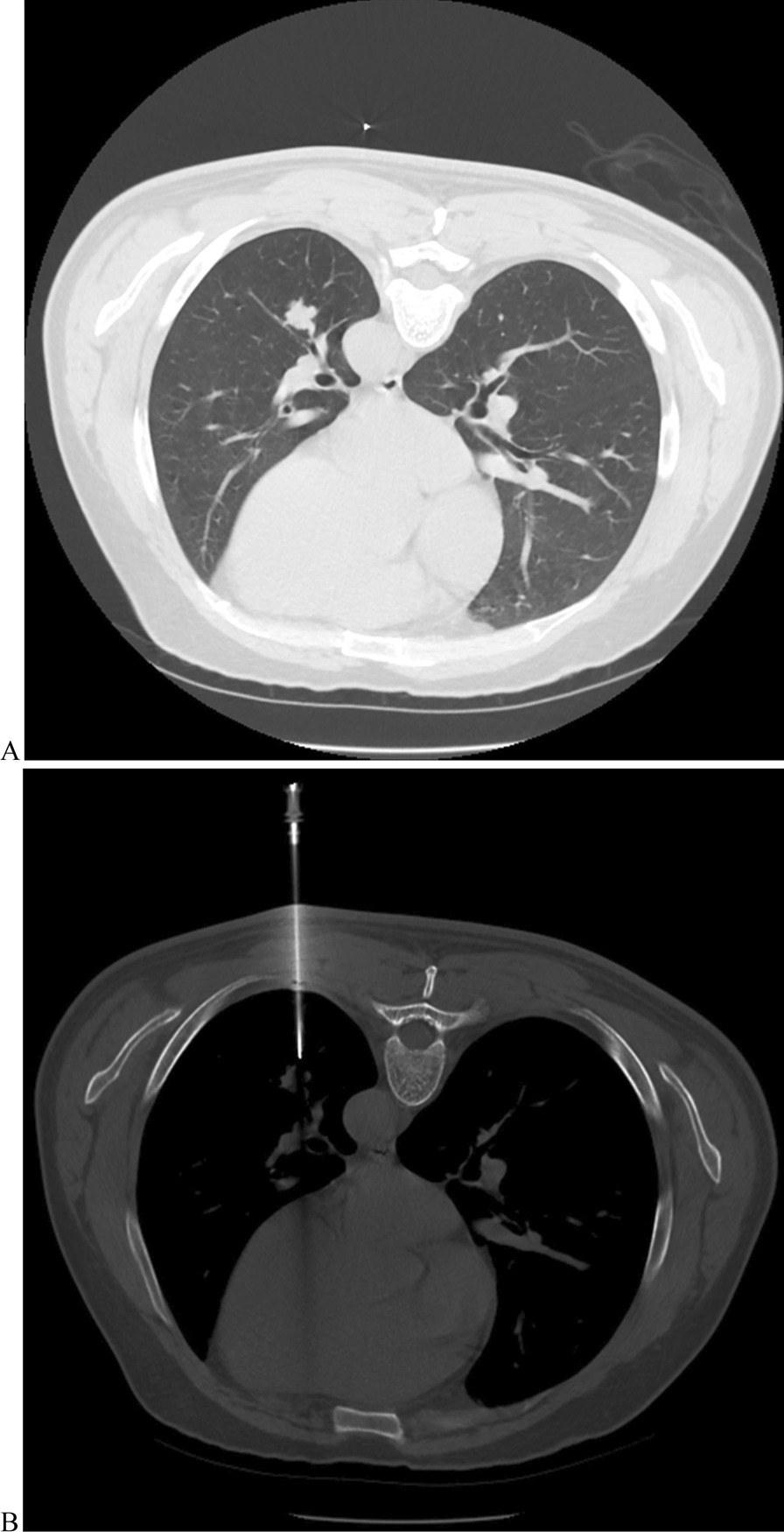
The images for standard-dose CT-guided biopsy for PN. **a** Preoperative CT for the PN; **b** the procedure of standard-dose CT-guided biopsy

Within the standard-dose group (Fig. 3), biopsy-based diagnoses included malignancy (n = 61), suspicious malignancy (n = 1), specific benignity (n = 4), and non-specific benignity (n = 34). Among them, the malignancy and specific benignity results could be approved as finalized- diagnoses. Suspicious malignancy was further validated as lung adenocarcinoma through surgical resection. Twenty-six non-specific benignities were confirmed as true benignities by CT medical observation (n = 22) or surgical resection (n = 4). Furthermore, 8 non-specific benignities were confirmed as false benignities by surgical resection. Therefore, the diagnostic yield, sensitivity, specificity, and diagnostic accuracy were 65%, 88.6%, 100%, and 92%, respectively.

**Fig. 4:**
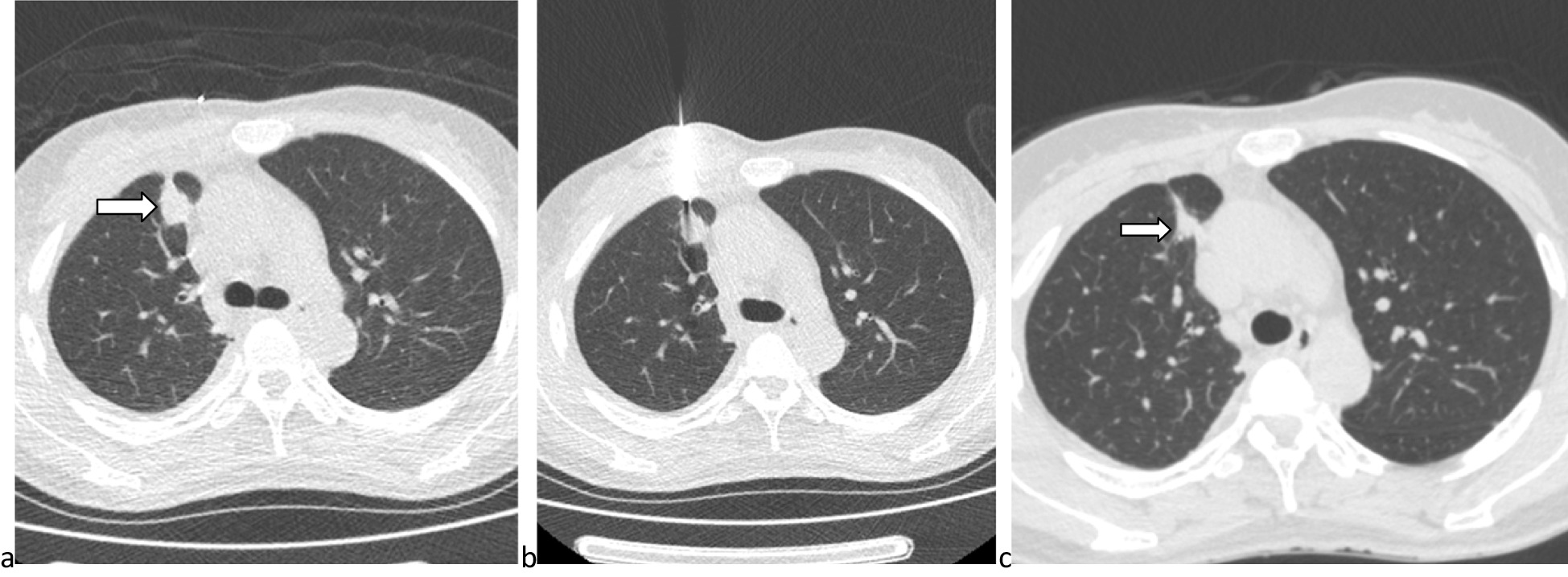
A group of images showed a case of true negative. **a** A PN (arrow) located at the right upper lobe; **b** The CT-guided biopsy indicated the benign result; **c** The PN (arrow) decreased 3 months later

There were no significant differences observed in diagnostic yield (P = 0.653), sensitivity (P = 0.554), and diagnostic accuracy (P = 0.579) between the two groups (Table 3). The number of true positive, true negative (Fig. 4), false positive, and false negative was shown in Table 3.

**Table 3:** Diagnostic performance between 2 groups

	Low-dose group (n = 100)	Standard-dose group (n = 100)	P value
Technical success rate	100%	100%	–
*Biopsy pathological diagnosis*			0.976
Malignancy	64	61	
Suspected malignancy	1	1	
Specific benign	4	4	
Non-specific benign	31	34	
*Final diagnosis*			0.877
Malignancy	71	70	
Benign	29	30	
*Diagnostic performance*			0.830
True positive	65	62	
False positive	0	0	
True negative	29	30	
False negative	6	8	
Diagnostic yield	68/100 (68%)	65/100 (65%)	0.653
Sensitivity	65/71 (91.5%)	62/70 (88.6%)	0.554
Specificity	29/29 (100%)	30/30 (100%)	–
Overall accuracy	94/100 (94%)	92/100 (92%)	0.579

The risk factors of diagnostic failure were detected by multivariate logistic regression test based on all patients. In the univariate logistic regression test, non-prone position (P = 0.059) and upper lobe (P = 0.038) were found to be associated with diagnostic failure. However, when these 2 factors were put into the multivariate logistic regression test, no risk factor (P = 0.323 and 0.165, respectively) was associated with the diagnostic failure (Table 4).

**Table 4:** Predictors of diagnostic accuracy

Variables	Univariate analysis			Multivariate analysis		
	Odds ratio	95% CI	P value	Odds ratio	95% CI	P value
Age	1.033	0.981–1.089	0.218			
*Gender*						
Male	1					
Female	0.727	0.220–2.409	0.602			
Smoking history	1.728	0.577–5.179	0.328			
BMI	0.991	0.845–1.162	0.909			
Emphysema	1.786	0.592–5.390	0.303			
*Body position*						
Prone	1			1		
Non-prone	2.983	0.961–9.259	0.059	1.906	0.531–6.839	0.323
Lesion size	1.036	0.914–1.176	0.580			
*Lung sides*						
Right	1					
Left	0.705	0.227–2.183	0.544			
*Lobe*						
Non-upper	1			1		
Upper	3.539	1.071–11.699	0.038	2.592	0.675–9.947	0.165
*Lesion-pleura distance*						
< 30 mm	1					
≥ 30 mm	0.649	0.139–3.024	0.582			
*Needle-pleura angle*						
< 50 degrees	1					
≥ 50 degrees	1.834	0.229–14.687	0.568			
Number of samples	2.321	0.778–6.929	0.131			
Duration of procedure	1.007	0.900–1.125	0.908			
*CT protocol*						
Low-dose	1					
Standard-dose	1.362	0.455–4.080	0.581			

*BMI* body mass index, *CI* confidential interval, *CT* computed tomography

### Complications

Pneumothorax was observed in 14 (14%) and 13 (13%) cases for low-dose and standard-dose groups, respectively (P = 0.836). Among them, 4 (28.6%) and 3 (23.1%) patients required chest tube insertion (P = 0.745). In the univariate logistic regression test, lesion-pleura distance ≥ 30 mm (P = 0.021), more needle pathways (P = 0.006) and longer duration of the procedure (P = 0.033) were found to be associated with pneumothorax. However, when these 3 factors were put into the multivariate logistic regression test, more needle pathways (P = 0.012) was the only risk factor of pneumothorax (Table 5).

**Table 5:** Predictors of biopsy-related complications

Variables	Univariate analysis			Multivariate analysis		
	Odds ratio	95% CI	P value	Odds ratio	95% CI	P value
*Pneumothorax*						
Age	0.997	0.964–1.032	0.884			
*Gender*						
Male	1					
Female	1.328	0.580–3.045	0.502			
Smoking history	1.185	0.526–2.671	0.682			
BMI	0.920	0.810–1.044	0.196			
Emphysema	1.163	0.491–2.759	0.731			
*Body position*						
Prone	1					
Non-prone	0.491	0.197–1.223	0.126			
Lesion size	0.996	0.914–1.085	0.926			
*Lung sides*						
Right	1					
Left	0.733	0.318–1.693	0.468			
*Lobe*						
Non-upper	1					
Upper	1.475	0.654–3.326	0.349			
*Lesion-pleura distance*						
< 30 mm	1			1		
≥ 30 mm	2.804	1.169–6.724	0.021	2.350	0.905–6.102	0.079
*Needle-pleura angle*						
< 50 degrees	1					
≥ 50 degrees	1.821	0.403–8.229	0.436			
Number of needle pathways	2.322	1.280–4.214	0.006	1.954	1.156–3.302	0.012
Duration of procedure	1.151	1.011–1.310	0.033	1.048	0.956–1.150	0.315
*CT protocol*						
Low-dose	1					
Standard-dose	0.918	0.408–2.067	0.836			
*High-grade lung hemorrhage*						
Age	0.982	0.951–1.014	0.261			
*Gender*						
Male	1					
Female	1.239	0.545–2.817	0.609			
Smoking history	0.366	0.148–0.905	0.030	0.425	0.156–1.159	0.095
BMI	1.117	1.000–1.248	0.050	1.111	0.979–1.260	0.103
Emphysema	0.728	0.292–1.817	0.497			
*Body position*						
Prone	1					
Non-prone	1.947	0.871–4.353	0.104			
Lesion size	0.802	0.701–0.927	0.002	0.931	0.850–1.020	0.123
*Lung sides*						
Right	1					
Left	0.817	0.361–1.848	0.628			
*Lobe*						
Non-upper	1			1		
Upper	2.252	0.995–5.098	0.052	2.085	0.812–5.351	0.127
*Lesion-pleura distance*						
< 30 mm	1			1		
≥ 30 mm	10.147	2.835–36.321	< 0.001	3.058	1.106–8.461	0.031
*Needle-pleura angle*						
< 50 degrees	1			1		
≥ 50 degrees	0.185	0.050–0.686	0.012	0.419	0.130–1.350	0.145
Number of needle pathways	1.532	0.969–2.422	0.068	1.352	0.726–2.517	0.341
Duration of procedure	1.187	1.041–1.355	0.011	0.993	0.896–1.101	0.896
*CT protocol*						
Low-dose	1					
Standard-dose	1.000	0.450–2.223	1.000			

*BMI* body mass index, *CI* confidential interval, *CT* computed tomography

Lung hemorrhage was observed in 24 (24%) and 26 (26%) patients in low-dose and standard-dose groups, accordingly (P = 0.744) in low-dose and standard-dose groups, respectively. Among them, 14 (28.6%) and 14 (23.1%) patients experienced high-grade hemorrhage (P = 0.749). All patients with lung hemorrhage were managed with hemostasis. In the univariate logistic regression test, smoking history (P = 0.03), higher BMI (P = 0.05), smaller lesion size (P = 0.002), upper lobe (P = 0.052), lesion-pleura distance ≥ 30 mm (P < 0.001), and needle-pleura angle ≥ 50 degrees (P = 0.012), more needle pathways (P = 0.068), longer duration of procedure (P = 0.011) were found to be associated with high-grade hemorrhage. When these 8 factors were assessed in the multivariate logistic regression test, lesion-pleura distance ≥ 30 mm (P = 0.031) was the only risk factor for high-grade hemorrhage (Table 5).

## Discussion

This RCT assessed the feasibility, safety, and diagnostic ability between low- and standard-dose CT-guided biopsy in PNs. Although the quality of images under the standard-dose CT was significantly better, low-dose CT resulted in similar technical success rates, the number of needle pathways, and the duration of procedures compared to standard-dose CT. Furthermore, unlike the conventional diagnostic images, the images for biopsy procedures do not require meticulous details of the lesion, but adequately observable locations of the needle tip and lesion are needed [12]. These findings may indicate that low-dose CT images made by our parameters can also fulfill the biopsy criteria for PNs.

Diagnostic yield usually indicates the ability to make a definite diagnosis by biopsy [16–18]. We found that the low-dose CT did not reduce the diagnostic yield of CT-guided biopsy. Furthermore, the diagnostic yield rates in both groups (68% and 65%) were similar to previous reports regarding CT-guided biopsy for PNs [9, 17]. Similarly, Shpilberg et al. [16] revealed that low-dose CT did not reduce diagnostic yield for spine biopsies (low-dose group: 69%; standard-dose: 60%, P = 0.60).

Diagnostic accuracy was the primary endpoint for this RCT. The low-dose group's sensitivity and diagnostic accuracy rates were comparable to the standard-dose group. This finding was similar to that in previous studies which compared the effectiveness of low-dose and standard-dose CT-guided lung biopsy [9, 10, 12–14]. Additionally, in concurrence with previous studies, the diagnostic accuracy rates in both groups (94% and 92%) were similar (90%-96%) for CT-guided biopsy for PNs [17, 19, 20]. However, we did not find any risk factors associated with the diagnostic failure. In past investigations on CT-guided biopsy, the risk factors of diagnostic failure usually encompassed fewer sample tissues and larger lesion size [9, 12, 21, 22]. We used the co-axial technique in this study and obtained 3–4 samples from each PN. Therefore, the number of sample tissues did not interfere with the diagnostic accuracy. With a larger lesion size, especially more than 5 cm, the diagnostic failure occurs due to the higher rates of obtained necrosis tissue [21]. Our RCT focused on the PNs, and we also found that lesion size was not associated with diagnostic failure at univariate logistic analysis (P = 0.580).

Biopsy-related complications were also the important endpoints in this RCT. The comparative results of pneumothorax and lung hemorrhage indicated that the low-dose protocol did not decrease the safety of biopsy. Furthermore, the chest tube requirement rates were only 4% and 3% in low-dose and standard-group. Risk factors for pneumothorax and high-grade hemorrhage included a greater number of needle pathways, smaller lesion size, and lesion-pleura distance ≥ 30 mm. These risk factors were consistent with past investigations regarding CT-guided lung biopsy [10, 20].

The low-dose CT protocol could be achieved by reducing the tube voltage and/or current [12, 23, 24]. In our study, we adjusted the tube current to 10% of the normal tube current (150 mA) and achieved a significant reduction in radiation exposure. This result was largely consistent with past investigations regarding low-dose CT-guided lung biopsy [12, 23, 24]. Although major dose reductions exacerbated noise and decreased image quality, low-dose CT at 120 kV and 15 mA produced an image quality adequate for the biopsy procedure. This result may be attributed to the use of iterative reconstruction technique [25]. The reconstruction technique can significantly reduce the image noise and provide better overall image quality [25].

This study had some limitations. First, we only used the block randomization method, but the randomization was not performed by the stratification of the lesion size. Therefore, the unbalanced data of lesion size was observed, which may cause selective bias. However, this study included PNs only. The difference in mean lesion size between the two groups was not large (24.8 mm vs. 23.5 mm). These findings may reduce the risk of bias. Second, this investigation did not find the risk factor of diagnostic failure. This finding could be due to the limited sample size. Third, we did not collected the CTDI_vol_ value. Although many previous studies also did not provide the CTDI_vol_ value [9, 10, 12, 16], DLP with CTDI_vol_ may have a better convincingness on the reduction of radiation dose. Fourth, the patients were from a single-center and further multi-center studies should be conducted to validate the results.

## Conclusions

In conclusion, compared to the standard-dose CT-guided biopsy for PNs, low-dose CT can significantly reduce radiation dose, while yielding comparable safety and diagnostic accuracy.

## Data Availability

The data that support the findings of this study are available from the corresponding author upon reasonable request.

## Abbreviations

CT: Computed tomography
ITT: Intention-to-treat
PN: Pulmonary nodule
PP: Per-protocol
RCT: Randomized controlled trial
